# Intestinal fatty acid binding protein is a disease biomarker in paediatric coeliac disease and Crohn’s disease

**DOI:** 10.1186/s12876-022-02334-6

**Published:** 2022-05-23

**Authors:** Michael Logan, Mary MacKinder, Clare Martha Clark, Aikaterini Kountouri, Mwansa Jere, Umer Zeeshan Ijaz, Richard Hansen, Paraic McGrogan, Richard K. Russell, Konstantinos Gerasimidis

**Affiliations:** 1 Human Nutrition, School of Medicine, Dentistry and Nursing, College of Medical, Veterinary and Life Sciences, University of Glasgow, New Lister Building, Glasgow Royal Infirmary, Glasgow, G31 2ER UK; 2 Department of Paediatric Gastroenterology, Royal Hospital for Children, Glasgow, UK; 3 Civil Engineering, School of Engineering, University of Glasgow, Oakfield Avenue, Glasgow, UK; 4 Department of Paediatric Gastroenterology, Royal Hospital for Sick Children, 9 Sciennes, Road, Edinburgh, EH9 1LF UK

**Keywords:** Crohn’s disease, Ulcerative colitis, Inflammatory bowel disease, Coeliac disease, Biomarkers, Paediatric

## Abstract

**Background:**

There is a clinical need to develop biomarkers of small bowel damage in coeliac disease and Crohn’s disease. This study evaluated intestinal fatty acid binding protein (iFABP), a potential biomarker of small bowel damage, in children with coeliac disease and Crohn’s disease.

**Methods:**

The concentration iFABP was measured in plasma and urine of children with ulcerative colitis, coeliac disease, and Crohn’s disease at diagnosis and from the latter two groups after treatment with gluten free diet (GFD) or exclusive enteral nutrition (EEN), respectively. Healthy children (Controls) were also recruited.

**Results:**

138 children were recruited. Plasma but not urinary iFABP was higher in patients with newly diagnosed coeliac disease than Controls (median [Q1, Q3] coeliac disease: 2104 pg/mL 1493, 2457] vs Controls: 938 pg/mL [616, 1140], *p* = 0.001). Plasma or urinary iFABP did not differ between patients with coeliac on GFD and Controls. Baseline iFABP in plasma decreased by 6 months on GFD (6mo GFD: 1238 pg/mL [952, 1618], *p* = 0.045). By 12 months this effect was lost, at which point 25% of patients with coeliac disease had detectable gluten in faeces, whilst tissue transglutaminase IgA antibodies (TGA) continued to decrease. At diagnosis, patients with Crohn’s disease had higher plasma iFABP levels than Controls (EEN Start: 1339 pg/mL [895, 1969] vs Controls: 938 pg/mL [616, 1140], *p* = 0.008). iFABP did not differ according to Crohn’s disease phenotype. Induction treatment with EEN tended to decrease (*p* = 0.072) iFABP in plasma which was no longer different to Controls (EEN End: 1114 pg/mL [689, 1400] vs Controls: 938 pg/mL [616, 1140], *p* = 0.164). Plasma or urinary iFABP did not differ in patients with ulcerative colitis from Controls (plasma iFABP, ulcerative colitis: 1309 pg/mL [1005, 1458] vs Controls: 938 pg/mL [616, 1140], *p* = 0.301; urinary iFABP ulcerative colitis: 38 pg/mg [29, 81] vs Controls: 53 pg/mg [27, 109], *p* = 0.605).

**Conclusions:**

Plasma, but not urinary iFABP is a candidate biomarker with better fidelity in monitoring compliance during GFD than TGA. The role of plasma iFABP in Crohn’s disease is promising but warrants further investigation.

*Trial registration*: Clinical Trials.gov, NCT02341248. Registered on 19/01/2015.

**Supplementary Information:**

The online version contains supplementary material available at 10.1186/s12876-022-02334-6.

## Introduction

Coeliac disease alongside inflammatory bowel disease (IBD) which includes both Crohn’s disease and ulcerative colitis are chronic diseases of the gastrointestinal tract (GIT) [1]. These diseases share similar presenting clinical symptoms including weight loss, malnutrition, and abdominal pain [1, 2].

The gold standard for assessment of intestinal damage in coeliac disease and IBD remains endoscopy coupled with biopsy [3, 4]. However, as endoscopy is an invasive procedure, routine assessment of disease activity of these diseases relies upon patient self-reported symptoms, along with blood and faecal inflammatory biomarkers which often do not reflect histological damage or recovery following therapy [5–8]. Faecal calprotectin (FC) has now become the mainstream marker of bowel inflammation in patients with IBD [9, 10]. However, there are conflicting reports surrounding the utility of FC in assessing intestinal inflammation in patients with isolated small bowel Crohn’s disease [11, 12]. In patients with coeliac disease, antibody tests such as the anti-transglutaminase antibodies (TGA), may not accurately reflect ongoing small bowel inflammation, due in part to the long half-life they take to decrease following introduction of a gluten-free diet (GFD) [13]. Currently, there are no reliable or specific laboratory biomarkers to assess inflammation within the small bowel in patients with coeliac disease or IBD, particularly Crohn’s disease.

Intestinal fatty acid binding protein (iFABP) is a small 5 kDa protein accounting for 1–2% of total cytosolic protein within enterocytes [14]. The tissue specificity of iFABP, as well its ability to be measured in readily available non-invasive samples (e.g. urine) make it an attractive candidate biomarker of tissue involvement/damage in the upper GIT.

Previous reports have shown that patients with IBD and coeliac disease have significantly higher concentration of circulating iFABP compared with healthy controls (Controls) but evidence has not always been consistent (Additional file 1: Table 1) [15–27]. The concentration of iFABP in patients with Crohn’s disease has also been shown to correlate with the pro-inflammatory cytokine tumour necrosis factor (TNF)-α and significantly decrease during treatment with anti-TNF-α therapy [20].

**Table 1:** Baseline anthropometry, faecal calprotectin concentrations and urinary and plasma concentrations of iFABP

	Controls (n = 28)	Established coeliac (n = 40)	Newly diagnosed coeliac (n = 12)	Crohn's disease (n = 47)	Ulcerative colitis (n = 11)
Female (%)	10/28 (35.7%)	23/40 (57.5%)	8/12 (66.7%)	13/47 (27.7%)	4/11 (36.4%)
Age (yrs)	9.1 (4.6, 11.7)	9.5 (7.2, 11.3)	11.0 (9.2, 12.9)	14.0 (11.2, 14.9)***	13.6 (11.4, 15.0)***
Height (m)	1.39 (1.08, 1.51)	1.36 (1.24, 1.48)	1.36 (1.32, 1.56)	1.53 (1.39, 1.64)***	1.59 (1.42, 1.67)**
Height (z-score)	0.64 (− 0.72, 1.14)	− 0.14 (− 0.66, 0.63)	0.02 (− 0.87, 0.62)	− 0.24 (− 0.69, 0.6)*	0.24 (− 0.51, 0.37)
Weight (kg)	35.15 (18.8, 47.2)	30.0 (22.2, 43.2)	33.5 (23.1, 40.9)	36.5 (30.0, 49.9)	48.4 (30.2, 62.1)**
Weight (z-score)	0.63 (− 0.39, 1.44)	− 0.03 (− 0.68, 0.66)	− 0.14 (− 0.82, 0.42)	− 0.74 (− 1.46, 0.35)***	0.62 (− 1.26, 0.89)
BMI (kg/m^2^)	16.7 (16.0, 20.3)	16.9 (15.0, 18.7)	17.1 (15.0, 19.0)	16.4 (14.9, 18.6)	18.1 (16.1, 23.4)
BMI (z-score)	0.35 (− 0.22, 1.19)	0.09 (− 0.58, 0.88)	0.06 (− 0.92, 0.64)	− 0.8 (− 1.74, 0.2)***	0.49 (− 1.29, 1.07)
CRP (mg/L)	–	–	–	9.0 (3.0, 26.5)	3.0 (2.5, 3.0)
TGA (U/mL)	–	1.9 (1.1, 6.5)	101.5 (32.8, 128.0)	–	–
GIP (μg/g)	–	0.16 (0.16, 0.16)	2.67 (1.0, 5.0)	–	–
Faecal calprotectin (mg/kg)	5.3 (2.9, 16.4)	22.1 (11.2, 47)	36.5 (25.0, 57.8)	1609 (1125, 1867)***	1581 (473, 1777)***
Urinary iFABP (pg/mg)	51 (27, 109)	–	26 (0, 80)	95 (32, 161)	38 (29, 81)
Plasma iFABP (pg/mL)	938 (616, 1140)	1070 (760, 1415)	2104 (1493, 2457)**	1339 (895, 1969)**	1309 (1005, 1458)

Comparisons are made compared with healthy controls

BMI, body mass index; CRP, C-reactive protein; TGA, IgA tissue transglutaminase antibodies; GIP, gluten immunogenic peptides; iFABP, intestinal fatty acid protein; yrs, years. Urinary iFABP measurements are expressed after urinary creatinine correction

*p < 0.05, **p < 0.01, ***p < 0.001

In patients with coeliac disease, serum iFABP concentrations have been shown to positively correlate with histological damage using the Marsh grading system [17]. More recently, iFABP has been proposed as a useful adjunct when used in conjunction with TGA levels as a non-invasive diagnostic tool for coeliac disease [16], whereas a Dutch study has also shown that circulating iFABP concentrations decreased rapidly when patients with coeliac disease were advised to follow a GFD [21].

There is currently limited literature which has explored responses of iFABP to therapy in patients with Crohn’s disease and coeliac disease and whether these biomarkers might be useful for monitoring changes in small bowel disease activity, and as biomarkers of GFD compliance in the latter group. Furthermore, the value of urinary compared to blood measurements of iFABP, with the former matrix potentially allowing non-invasive and remote monitoring of disease activity, has only been explored in a recent pilot study in adult patients with Crohn’s disease [15].

The current study measured plasma and urinary iFABP levels in paediatric patients with Crohn’s disease, ulcerative colitis, and coeliac disease at diagnosis, and followed patients with Crohn’s and coeliac disease through treatment with exclusive enteral nutrition (EEN) or GFD, respectively. We compared results against groups of Controls. Our hypothesis was that, at disease presentation, iFABP levels will be higher in patients with coeliac disease and Crohn’s disease and that their levels will decrease during therapy.

## Method

### Study design and recruitment

Patients with coeliac disease were recruited between August 2011 and September 2013. Diagnosis of coeliac disease was based on the contemporary British Society for Paediatric Gastroenterology Hepatology and Nutrition (BSPGHAN) guidelines with all patients receiving small bowel endoscopy and biopsy [4]. Two cohorts of patients with coeliac disease were enrolled in the current study: (a) *longitudinal cohort* of newly diagnosed patients with coeliac disease with blood, faecal and urine samples collected at disease diagnosis and again at 6- and 12-months following recommendation to follow a GFD; and (b) a separate *cross-sectional* group of patients with previously diagnosed coeliac disease on recommended treatment with GFD. A single collection of blood and faecal samples was from these children at time of study recruitment.

Patients undergoing investigation for diagnosis of IBD were recruited between October 2014 to May 2017. Diagnosis of IBD was based on the European Society for Paediatric Gastroenterology Hepatology and Nutrition (ESPGHAN) criteria [28]. Patients with Crohn’s disease diagnosis were treated with 8-week course of EEN as per guidelines [29, 30]. Anthropometry, blood, faecal and spot urine samples were obtained before the start of treatment and again at the end of EEN. A third group was formed of patients who were subsequently diagnosed with ulcerative colitis. Since ulcerative colitis is limited to the colon and iFABP is a biomarker of small bowel damage, we hypothesised that this group of patients would present a lower concentration of iFABP in blood or in urine compared to patients with coeliac disease or Crohn’s disease at diagnosis. The clinical phenotype of all patients with IBD was determined according to Paris criteria and with consideration of baseline macroscopic endoscopic, histological and radiological investigations [31].

Plasma samples were also obtained from a group of children who were admitted to the same hospital for acute diagnostic investigations but following review did not present any significant pathology, had normal haematology, no inflammatory response, and no need for follow-up reviews up to six months, as this was ascertained via their electronic medical records. This group was labelled as Controls. For urinary analysis, samples were collected from a further cohort of Controls were recruited from the local community through advertisement to act as a control group for measurements on iFABP in urine.

**Fig. 1:**
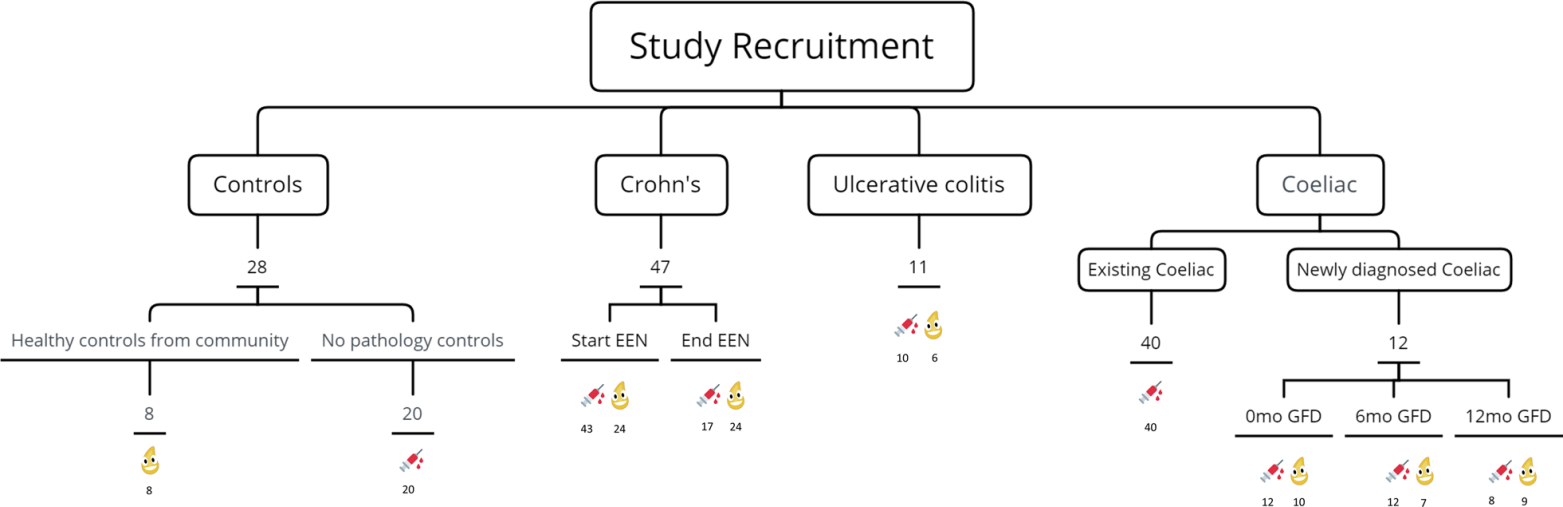
Schematic representation of study recruitment with sample type collection in each study group. Abbreviations mo: month, GFD: gluten-free diet

A schematic of patients and sampling timepoints is represented in Fig. 1. The study was approved by the NHS West of Scotland Research Ethics Committee (14/WS/1004 for Crohn’s disease patients and 11/WS/0006 for patients with coeliac disease). Written informed consent was taken from participants and their carers according to good clinical practice. The trial was registered on clinicaltrials.gov with the identifier NCT02341248 on 19/01/2015. All methods were carried out in accordance with relevant guidelines and regulations.

### Measurements of iFABP

Whole blood was collected in lithium heparin vacutainer tubes. Plasma was separated within two hours of collection and stored at − 70 °C until analysis. Plasma and urinary iFABP concentrations were determined using the Quantikine anti-Human-iFABP (DFBP20) (R&D Systems Bio-Techne) ELISA kit according to the manufacturer’s instructions. To account for a dilution effect in the concentration of iFABP in urine, results were expressed as ratio to urinary creatinine [32].

### Disease activity markers in patients with Crohn’s disease

Clinical disease activity in patients with Crohn’s disease was assessed using the weighted paediatric Crohn’s disease activity index (wPCDAI) [33]. A wPCDAI score between 12.5 to 39.5, 40 to 57, and > 57.5 was classified as mild, moderate, and severe disease activity, respectively. Faecal calprotectin concentration was determined using the CALP0170 (ALP) (CalproLab™, Lysaker, Norway) as previously described [34]. A cut-off value below 250 mg/kg was considered normal.

### Biomarker of recent gluten intake in patients with coeliac disease

Faecal gluten immunogenic peptides (GIP), an objective marker of ingestion of gluten the past 2–3 days, was measured in faeces from children with coeliac disease using the iVYLISA GIP Stool ELISA kit (Biomedal, Spain) as previously described [35]. Patients were deemed to be compliant to GFD if their concentration of GIP was < 0.156 μg/g (lower detection limit of the assay).

### Statistics

Continuous data are presented as medians (IQR) unless otherwise stated. To compare differences in urinary or plasma iFABP concentrations between disease groups, a general linear model was used with the Fisher least significant difference correction test for post-hoc comparisons. To compare longitudinal changes in iFABP concentrations either through GFD in patients with coeliac disease, or during EEN in patients with Crohn’s disease, Wilcoxon signed-rank tests were utilised to account for the subject effect.

Correlations were explored with Spearman rank correlation. Statistical analysis was performed using Minitab version 18 statistical software (Minitab Ltd). *p* values below 0.05 were considered statistically significant. Multiple testing was corrected using the Tukey test.

## Results

### Participant’s characteristics

In total, 138 children (females: 58/138, 42%; age: 11.2 yrs [Q1: 8.5, Q3: 14.0]) were recruited, providing a total of 88 urinary and 162 plasma samples, Fig. 1 and Table 1. Few patients were lost in follow up and for some others all biological samples (urine, blood and stool) were not provided by the participants, or extra blood volumes for research tests could not be obtained (Fig. 1). Blood samples from Controls were collected from younger children than the other groups. Nonetheless, there was no significant association between age and the concentration of iFABP in urine and in blood within the healthy control groups, suggesting an absence of a biological variation with age.

### iFABP concentrations in patients with coeliac disease and changes during GFD

**Fig. 2:**
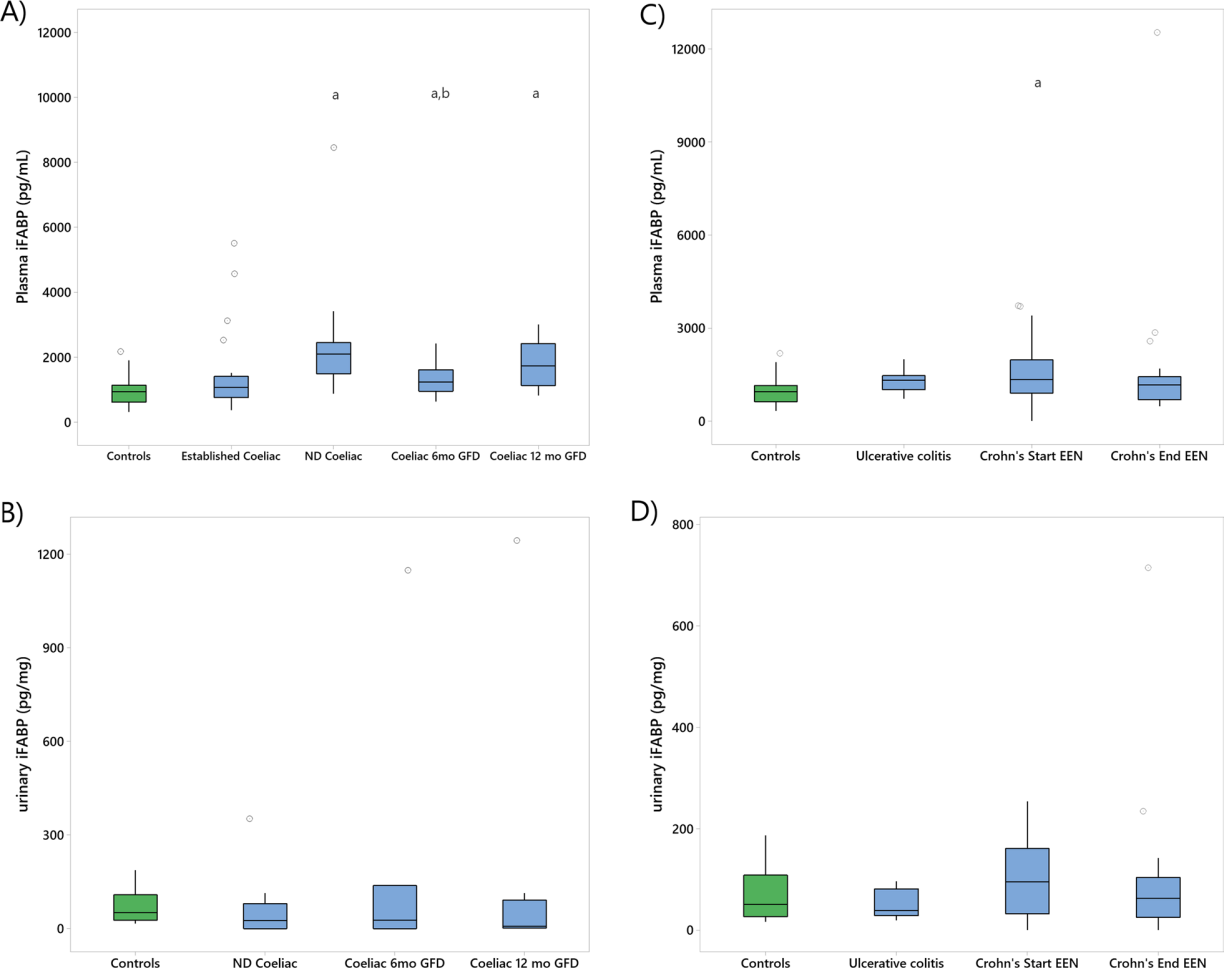
Boxplots **A** plasma, **B** urinary iFABP concentrations in Controls and patients with Coeliac during GFD; **C** plasma, **D** urinary iFABP concentration in Controls and patients with Colitis and Crohn’s during exclusive enteral nutrition. Coeliac: coeliac disease, ND: newly diagnosed, Crohn’s: Crohn’s disease, Controls: healthy control, mo: month, GFD: gluten-free diet, EEN: exclusive enteral nutrition. Urinary iFABP measurements are expressed after urinary creatinine correction, a: *p* < 0.05 compared with Controls; b: *p* < 0.05 compared with ND Coeliac

Plasma iFABP concentration was significantly higher in newly diagnosed coeliac disease compared to Controls (plasma iFABP newly diagnosed coeliac disease: 2104 pg/mL [1493, 2457] vs Controls: 938 pg/mL [616, 1140], *p* = 0.001), and patients with established coeliac disease (plasma iFABP newly diagnosed coeliac disease: 2104 pg/mL [1493, 2457] vs established coeliac disease: 1070 pg/mL [760, 1415], *p* = 0.002), Fig. 2. There was no significant difference in plasma iFABP levels between patients with established coeliac disease and Controls (plasma iFABP established coeliac disease: 1070 pg/mL [760, 1415] vs Controls: 938 pg/mL [616, 1140], *p* = 0.41), Table 1.

Following coeliac disease diagnosis and GFD recommendation, median plasma TGA levels significantly decreased after 6 months of GFD (plasma TGA at 6mo GFD: 9 U/mL [3, 11] vs newly diagnosed coeliac disease: 102 U/mL [33, 128]; *p* = 0.006) with a further significant decrease observed between 6 and 12 months on GFD (plasma TGA at 12mo GFD: 5 U/mL [2, 7] *p* = 0.022), Fig. 2.

During the same period, plasma iFABP levels decreased significantly from baseline, by 6 months GFD (plasma iFABP at 6mo GFD: 1238 pg/mL [952, 1618] vs newly diagnosed coeliac disease GFD: 2104 pg/mL [1493, 2457] *p* = 0.045), Table 1. In contrast to TGA pattern, by 12 months this effect was lost (plasma iFABP at 12mo GFD: 1733 pg/mL [1134, 2422] vs newly diagnosed coeliac disease: 2104 pg/mL [1493, 2457] *p* = 0.265), Table 1. Yet patients at 6mo GFD and at 12mo GFD had significantly higher levels of plasma iFABP compared to Controls (plasma iFABP 6mo GFD: 1238 pg/mL [952, 1618] vs Controls: 938 pg/mL [616, 1140], *p* = 0.041; 12mo GFD: 1733 pg/mL [1134, 2422] vs Controls: 938 pg/mL [616, 1140], *p* = 0.001), Table 1. During the same timeframe, the proportion of patients with coeliac disease with positive measurements of faecal GIP, indicating recent intake of gluten, was 100% (9/9), 20% (2/10), 25% (2/8) at diagnosis, 6- and 12-months post-diagnosis respectively.

In urine samples, iFABP levels did not differ significantly between patients newly diagnosed with coeliac disease and Controls (urinary iFABP Controls: 50.9 pg/mg [26.6, 108.6] vs newly diagnosed coeliac disease: 25.6 pg/mg [0.0, 80.0], *p* = 0.904) and its concentration did not change following GFD recommendation either at 6 or 12 months (urinary iFABP at coeliac disease diagnosis: 26 pg/mg [0, 80] vs 6mo GFD: 27 pg/mg [0, 138] *p* = 0.40; coeliac disease diagnosis vs 12mo GFD: 8 pg/mg [2, 92], *p* = 0.51).

After pooling data across all coeliac disease groups, a significant positive correlation was observed between plasma iFABP levels with TGA (rho: 0.40, *p* = 0.001) and with GIP levels (rho: 0.44, *p* < 0.001). There was also a significant positive correlation between urinary iFABP with GIP levels (rho: 0.47, *p* = 0.045) but not for TGA titres levels (rho: 0.347, *p* = 0.113).

### iFABP in patients with Crohn’s disease and changes during exclusive enteral nutrition

Prior to EEN initiation, patients with Crohn’s disease had significantly higher plasma iFABP concentrations than Controls (plasma iFABP EEN Start: 1339 pg/mL [895, 1969] vs Controls: 938 pg/mL [616, 1140], *p* = 0.008). There was no significant difference in plasma iFABP between patients with ulcerative colitis compared with Controls (plasma iFABP ulcerative colitis: 1309 pg/mL [1005, 1458] vs Controls: 938 pg/mL [616, 1140], *p* = 0.301). In urine, patients with Crohn’s disease and ulcerative colitis had similar iFABP concentrations as compared to Controls (urinary iFABP EEN Start: 95 pg/mg [32, 161] vs Controls: 52 pg/mg [27, 109], *p* = 0.541; ulcerative colitis: 38 [29, 81] vs Controls: 52 pg/mg [27, 109], *p* = 0.651), Table 1.

All but one of the patients (46/47) with Crohn’s disease were treated with EEN. Of these, 37 (80%) completed an 8-week course with EEN. Nine patients who did not complete EEN either experienced worsening of symptoms within the first week of EEN initiation or could not tolerate EEN and were subsequently treated with corticosteroids. Amongst all patients who started on EEN, 29/46 (63%) entered clinical remission (wPCDAI < 12.5). In the group which completed EEN, baseline FC levels decreased significantly (FC Start EEN: 1608 mg/kg [1101, 1801] vs End EEN: 516 mg/kg [257, 1756]; *p* = 0.002).

In samples collected prior to EEN initiation, plasma iFABP was significantly higher in patients to successfully complete 8wk EEN than patients who did not complete EEN (plasma iFABP complete 8wk EEN: 913 pg/mL [731, 1172] vs did not complete 8wk EEN: 1445 pg/mL [999, 1979], *p* = 0.038). There was no significant difference in plasma iFABP concentration, based on clinical disease activity at time of treatment initiation (plasma iFABP wPCDAI mild disease: 1249 pg/mL [830, 1858], wPCDAI moderate disease: 1209 pg/mL [883, 1505], wPCDAI severe disease: 1448 pg/mL [926, 2802], *p* = 0.3).

Neither plasma nor urinary iFABP differed significantly between patients according to their disease location phenotype (plasma iFABP colonic disease: 1129 pg/mL [869, 1515] vs non-colonic disease: 1445 pg/mL [1040, 2419], *p* = 0.073; urinary iFABP colonic disease: 61 pg/mg [33, 120] vs non-colonic disease: 125 pg/mg [19, 229] *p* = 0.196), or upper GIT involvement (plasma iFABP upper GIT: 1436 pg/mL [1146, 1941] vs no upper GIT: 1150 pg/mL [873, 1999] *p* = 0.863; urinary iFABP upper GIT: 95 pg/mg [40, 143] vs no upper GIT: 90 pg/mg [5, 208] *p* = 0.564).

**Fig. 3:**
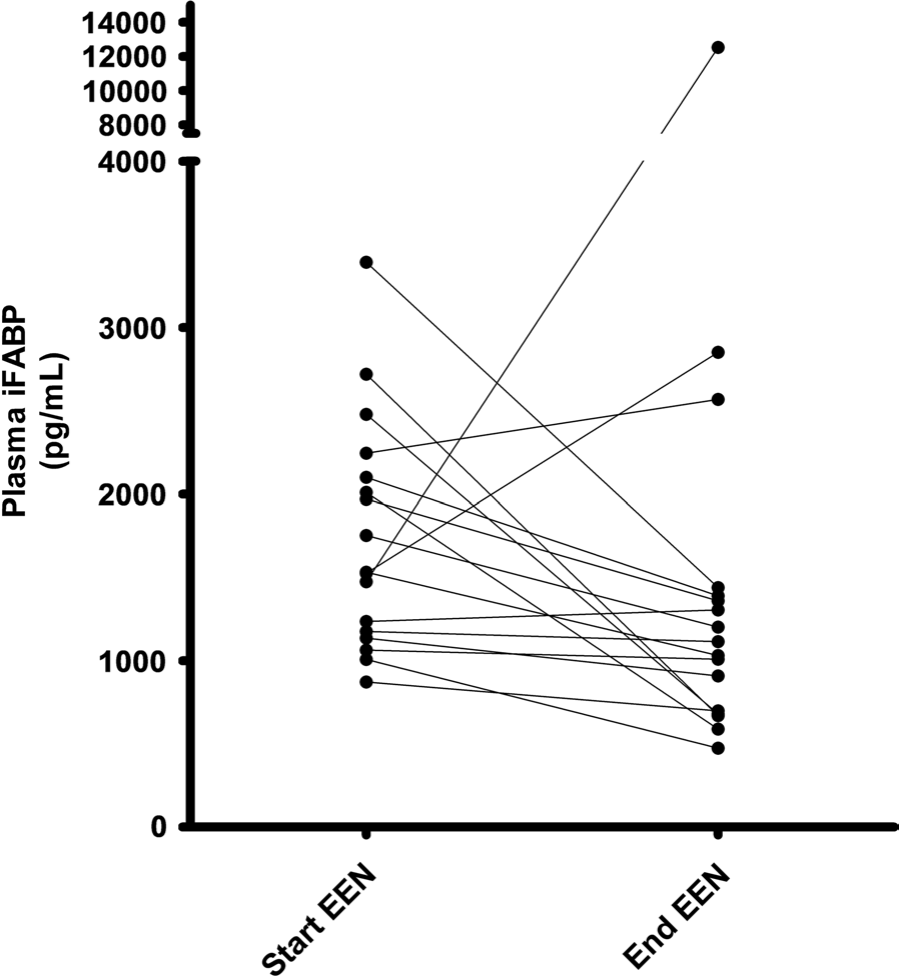
Individual value plot of pairwise plasma iFABP levels in patients with Crohn's during treatment with exclusive enteral nutrition

Paired urine samples were collected from 24 patients with Crohn’s disease prior to initiation of EEN and at the end of EEN. There was no significant change in urinary iFABP levels during EEN (EEN start: 101 pg/mg [38, 167] vs EEN end: 63 pg/mg [30, 93], *p* = 0.78). Likewise, paired plasma samples were available from 17 patients with Crohn’s disease to assess the change in iFABP concentration over the course of EEN. Plasma iFABP non-significantly decreased from baseline to the end of EEN (plasma iFABP EEN Start: 1532 pg/mL [1156, 2127] vs EEN End: 1114 pg/mL [689, 1400], *p* = 0.072), Fig. 3.

There was no significant difference in plasma or urinary iFABP levels from samples collected at the end of EEN compared with Controls (plasma iFABP End EEN: 1114 pg/mL [689, 1400] vs Controls: 938 pg/mL [616, 1140], *p* = 0.164; urinary iFABP End EEN: 63 pg/mg [30, 93] vs Controls: 52 pg/mg [27, 109], *p* = 0.751).

Despite a trend observed for plasma measurements, there was no significant difference in urinary or plasma iFABP concentrations according to FC levels at the end of EEN (FC < 250 mg/kg plasma iFABP: 629 pg/mL [503, 941] vs FC > 250 plasma iFABP: 1333 pg/mL [934, 2348], *p* = 0.06; FC < 250 mg/kg urinary iFABP: 26 pg/mg [9, 111] vs FC > 250 urinary iFABP: 69 pg/mg [45, 129], *p* = 0.25). Likewise, there was no significant correlation between either plasma or urinary iFABP levels and FC levels in patients with Crohn’s disease (plasma iFABP rho: 0.123, *p* = 0.467; urinary iFABP rho: -0.105, *p* = 0.668).

## Discussion

There still remains an unmet clinical need to identify non-invasive biomarkers in patients with coeliac disease and IBD, which are not only sensitive and specific enough to assess histological damage, but also offer dynamic response to recent changes in inflammatory state following treatment.

In the current study, we have shown the value of using plasma, but not urinary iFABP, as a potential non-invasive biomarker to complement differential diagnosis of coeliac disease and, importantly, for monitoring compliance with GFD. We were able to show that variation in compliance with GFD, as indicated by measurements of GIP in faeces, coincided with those of plasma iFABP, but not with TGA titres, within the same population. This observation suggests that while TGA might be an important biomarker to screen and diagnose coeliac disease, its usage for monitoring rapid changes in compliance with GFD is probably inferior to plasma iFABP. Indeed, with a half-life of 11 min while in circulation, plasma iFABP is likely to be a more sensitive biomarker of compliance to GFD when compared to TGA which can take up to two years to normalise [36, 37]. Adriaanse et al.(2017) showed that serum iFABP normalised in 82% of patients with coeliac disease by 26 weeks GFD. However, TGA levels had normalised in only 47% of patients. The same group showed that serum iFABP increased significantly in patients with coeliac disease following recent gluten challenge and had begun to significantly decrease within 14 days of return to GFD [23], demonstrating the dynamic changes of iFABP. While it is yet unknown what level of gluten consumption is required to elicit a subsequent increase in iFABP concentration, in this previous study, there was no significant difference in the degree of iFABP increase between patients who ingested 3 g gluten/day or 7.6 g gluten/day.

Our observations of higher plasma iFABP levels in patients with active Crohn’s disease compared with Controls corroborates with the available literature [20, 25].

Sarikaya et al. reported that patients with clinically active Crohn’s disease had significantly higher levels of serum iFABP compared with Controls as well as patients with Crohn’s disease in clinical remission [25]. Al-Saffar et al. also reported significantly higher serum iFABP in patients with Crohn’s disease compared with Controls, and further demonstrated that serum iFABP levels significantly decreased in clinical response to anti-TNFα therapy [20]. Like Al-Saffar et al., our results demonstrated a tendency for a significant decrease in plasma iFABP during EEN treatment. Furthermore, as there was no significant difference in plasma iFABP concentrations between samples collected at the end of EEN in patients with Crohn’s disease compared with Controls, one can argue that there was a decrease in plasma iFABP and improvement in small bowel damage during EEN, which we were unable to detect due inter-individual responses and the small sample.

Regarding the influence of disease location our results are in accordance with the largest study to date, which explored the utility of iFABP as a biomarker of disease activity in patients with Crohn’s disease (n = 128) [24]. In this previous study, the authors found no significant difference in plasma iFABP concentrations between patients with clinically active disease and others in clinical remission. Furthermore, circulating iFABP concentrations were not affected by disease location in patients with Crohn’s disease which we also observed in the present study. This finding is further supported by the absence of differences in urinary or plasma levels of iFABP between patients with Crohn’s disease, where small bowel involvement is common, and patients with Colitis where the condition is confined to the colon.

There have been a few published reports of urinary iFABP as a useful biomarker in helping to distinguish between patients with necrotising enterocolitis from septic patients, as well as in predicting acute mesenteric ischemia [38–40]. A recent pilot study reported that urinary iFABP may be a potentially useful biomarker of disease activity in adults with Crohn’s disease, as it decreased in concentration during treatment with EEN [15]. However, in the current study, urinary iFABP failed in predicting either disease type or changes in response to treatment.

The current study is limited by the small number of patients in some subgroup analysis, such as changes in iFABP levels in children with Crohn’s disease during EEN. Also, we did not have histological scoring in children with coeliac disease before and during treatment with GFD to associate with changes in iFABP levels. Ideally, plasma measurements of iFABP in Controls should have been obtained from healthy children in general community; however this was not allowed on ethical grounds at the time the study took place. Furthermore, GIP is a biomarker of recent gluten ingestion and unless serial measurements are available it cannot be used to assess long-term compliance to a GFD.

In conclusion, this study highlights the utility of iFABP measurements in plasma but not in urine, in helping in monitoring compliance during GFD, with potentially better performance when compared to other mainstream biomarkers. The role of plasma iFABP in Crohn’s disease and the effect of induction treatment might also be promising but warrants further investigation.

## Supplementary Information

**Additional file 1**. **Supplementary Table 1.** Evidence table of studies exploring the utility of intestinal fatty acid binding protein as a biomarker of disease activity in patients with coeliac disease and inflammatory bowel disease.

## Data Availability

The datasets used within the current study are available from the corresponding author on reasonable request.

## Abbreviations

IBD: Inflammatory bowel disease
GIT: Gastrointestinal tract
FC: Faecal calprotectin
TGA: Anti-transglutaminase antibodies
GFD: Gluten-free diet
iFABP: Intestinal fatty acid binding protein
Controls: Healthy controls
TNF: Tumour necrosis factor
EEN: Exclusive enteral nutrition
BSPGHAN: British Society for Paediatric Gastroenterology Hepatology and Nutrition
ESPGHAN: European Society for Paediatric Gastroenterology Hepatology and Nutrition
wPCDAI: Weighted paediatric Crohn’s disease activity index
GIP: Gluten immunogenic peptides
