# Supplementary material for: Intestinal fatty acid binding protein is a disease biomarker in paediatric coeliac disease and Crohn’s disease
Source: BMC Gastroenterol. 2022 May 23;22:260. doi: 10.1186/s12876-022-02334-6 (PMC9125891; doi:10.1186/s12876-022-02334-6)
Supplement: Supplementary file 1 — Additional file 1. Supplementary Table 1. Evidence table of studies exploring the utility of intestinal fatty acid binding protein as a biomarker of disease activity in patients with coeliac disease and inflammatory bowel disease. [file 12876_2022_2334_MOESM1_ESM.docx]

Supplementary Table 1 Evidence table of studies exploring the utility of intestinal fatty acid binding protein as a biomarker of disease activity in patients with coeliac disease and inflammatory bowel disease

| Study | Study participants | Study age group | Subject characteristics | Methods | Results |
| --- | --- | --- | --- | --- | --- |
| Ho 2020^[15]^ | Explore changes in urinary iFABP & L-FABP concentrations in patients with Crohn’s during treatment with EEN | Adults | Crohn’s: 14 | Urinary iFABP & L-FABP (Hycult Biotech),  Urinary CLDN-3,  CLDN-3,  BMI,  HBI,  CDAI,  serum IGF-1,  CRP,  ESR,  FC | Urinary iFABP decreased significantly during EEN (median [Q1, Q3], 0wk EEN: 55.7 ng/g [15.1, 84.1] vs 8wk EEN: 11.7 ng/g [7.8, 34.5], p=0.03)  No significant correlation between urinary iFABP & HBI, CDAI, serum IGF-1, CRP, ESR, or FC  Urinary L-FABP decreased significantly during EEN (median [Q1, Q3], 0wk EEN: 6.6 μg/g [3.8, 14.6] vs 11.1 μg/g [6.1, 22.0], p=0.03)  Positive correlation between L-FABP levels & serum IGF-1 levels (Spearman’s rho=0.60, p=0.02). No other significant correlation between urinary L-FABP and HBI, CDAI, CRP, ESR, or FC  No significant change in urinary CLDN-3 concentrations during EEN (median [Q1, Q3], 0wk EEN: 1.4 μg/g [1.1, 2.7] vs 8wk EEN: 1.3 μg/g [0.8, 2.1], p>0.05)  No significant correlation between urinary CLDN-3 & HBI, CDAI, serum IGF-1, CRP, ESR, or FC  Urinary calprotectin levels did not significantly change during EEN (median [Q1, Q3], 0wk EEN: 615.0 μg/g [194.9, 1,535] vs 8wk EEN: 591.1 μg/g [125.9, 1,811], p>0.05  Urinary calprotectin levels did not correlate with FC (Spearman’s rho=0.002, p=0.99)  No significant correlation between urinary calprotectin & HBI, CDAI, serum IGF-1, CRP, ESR, or FC |
| Oldenburger 2018^[16]^ | Group A:  Patients with suspected Coeliac (TGA between 1-10x ULN, and  expressed HLA DQ2 and/or DQ8)  Group B:  Controls with small stature | Paediatrics | Group A: 95 (Coeliac: 71, normal biopsy non-Coeliac: 24)  Group B: 161 Controls | Serum iFABP (Hycult Biotech),  Serum IgA TGA,  IgA EMA,  HLA-DQA1 and -DQB1 allele typing | 71 patients in group A subsequently diagnosed with Coeliac. The remaining 24 did not have Coeliac upon endoscopy  Histologically proven Coeliac patients had higher median iFABP concentrations vs HC (median [Q1, Q3], Coeliac: 725 pg/mL [Q1: 431, Q3: 1109 pg/mL] vs HC: 263 pg/mL [Q1: 141, Q3: 422], P<0.0001)  Median iFABP in patients with normal biopsy was significantly higher vs Controls (median [Q1, Q3], normal biopsy non-Coeliac: 497 pg/mL [Q1: 357, Q3: 826] vs HC, P<0.0001)  No significant difference in median iFABP concentrations between Coeliac vs normal biopsy non-Coeliac (P=0.13)  Stratifying patients through combination of iFABP & TGA levels  11/24 (46%) of Coeliac patients could be correctly stratified using iFABP cut-off level of 880 pg/mL in patients with TGA≥50 U/mL  12/19 (63%) of Coeliac patients could be correctly stratified using iFABP cut-off level of 620 pg/mL in patients with TGA≥60 U/mL |
| Al-Saffar 2017 ^[20]^ | Longitudinal  Existing Crohn’s patients undergoing IFX infusion (5mg/kg/body weight) for maintenance of remission. Repeated serum samples obtained 1 wk post IFX infusion at 1wk, 3wk, 7wk) | Adults | Crohn’s: 10  Lactulose: mannitol healthy control: 31  Anti-TNFα healthy comparator group: 61 | Serum iFABP (Hycult Biotech),  Lactulose: mannitol ratio,  CRP,  ESR,  TNFα level,  IL6,  Immunohistochemistry,  HBI | Patients with Crohn’s had increased mean TNFα levels vs HC (mean [SD] Crohn’s: 2.34 ± 0.22 vs HC: 1.48 ± 0.06 ng/L, P<0.001)  Patients with Crohn’s had increased mean serum iFABP concentrations vs HC (mean [SD] Crohn’s: 2.07 ± 0.23 vs HC: 0.84 ± 0.13 μg/L, P<0.001) |
| Adriaanse 2017^[21]^ | Presenting with a clinical suspicion of Coeliac, elevated Coeliac autoantibody titres, and HLA-DQ2 and/or -DQ8 genotype | Paediatrics | Coeliac: 79  HC: 91 (children presenting with Coeliac symptoms, yet with normal TGA-IgA and IgA-EMA titres) | Plasma iFABP (in-house ELISA),  Coeliac autoantibody titres,  HLA genotype  TGA-IgA  Marsh grading | Previous ROC curve analysis on historical unpublished paediatric cohort (Coeliac [n=38] & control children [n=40]) identified optimal cut-off of 450 pg/mL to discriminate villous atrophy from normal intestinal mucosa (positive predictive value of 87.5% (sensitivity: 92.1%, specificity: 87.7%)  61/79 (77%) patients with Coeliac had serum iFABP concentrations above cut-off (450 pg/mL)  Patients with Coeliac had higher median iFABP concentrations vs HC (median [Q1, Q3], Coeliac: 726 pg/ml [Q1: 458, Q3: 1,024] vs HC: 218 pg/ml [Q1: 143, Q3: 323], P<0.001)  Median iFABP concentrations decreased significantly within 3wk of GFD (median [Q1, Q3], 0wk GFD: 854 pg/mL [Q1: 635, Q3: 1,265] vs 3wk GFD: 498 pg/mL [Q1: 270, Q3: 697], P<0.05)  Median iFABP levels in patients with Coeliac similar with Controls by 26wk GFD (median [Q1, Q3], 26wk GFD: 231 pg/mL [Q1: 185, Q3: 318])  iFABP concentrations decreased below the cut-off value in 42%, 68% (median [Q1, Q3], 382pg/mL [Q1: 253, Q3: 497]), 70% (274pg/mL [Q1: 207, Q3: 469]) and 82% (231pg/mL [Q1: 185, Q3: 318]) of patients with Coeliac after 3wk, 6wk, 12wk and 26wk GFD, respectively  Median TGA-IgA decreased significantly by 3wks GFD (median [Q1, Q3], 0wk GFD: 314 U/mL [Q1: 97, Q3: 1,025] vs 3wk GFD: 148 U/mL [Q1: 68, Q3: 363], P<0.05)  TGA-IgA titres remained elevated at six months GFD  Increased iFABP levels in patients with villous atrophy vs HC:  Marsh grade IIIC (median [Q1, Q3], 775 pg/mL [Q1: 482, Q3: 1,265] vs HC, P<0.001),  Marsh IIIB (876pg/mL [Q1: 583, Q3: 1,070] vs HC, P<0.001)  Marsh IIIA (567pg/mL [Q1: 466, Q3: 849] vs HC, P<0.001]  iFABP levels did not correlate with clinical symptoms at disease diagnosis or during GFD (P=0.511) |
| Uhde 2016^[22]^ | Aimed to determine whether sensitivity to wheat in the absence of Coeliac was associated with systemic immune activation | Adults | Coeliac: 40,  Non-Coeliac wheat sensitivity (NCWS): 80 non-restrictive diet (20: before & after restrictive diet),  Controls: 40 | Serum iFABP (R&D Systems),  IgA TGA,  IgG & IgA antibodies to DGP,  LBP,  Soluble CD14 (sCD14) | Median serum iFABP significantly higher in patients with NCWS & Coeliac vs Controls (P<0.0001 for both)  Within the NCWS group, serum iFABP concentrations correlated with LBP (r=0.360, P=0.001), sCD14 (r=0.461, P<0.0001), EndoCAb IgM (r=0.305, P=0.003) & anti-flagellin IgM antibody reactivity (r=0.239, P=0.033)  Serum iFABP concentrations significantly correlated with IgA TGA (r=0.559, P<0.0001) in patients with Coeliac |
| Adriaanse 2016^[23]^ | Measure changes in serum iFABP concentrations in patients with Coeliac during 14d gluten challenge | Adults | Coeliac: 20 (High gluten challenge [7.6 g/d gluten]: 10, Low gluten challenge [3.6 g/d gluten]: 10),  Controls: 43 | Serum iFABP (Quanta Lite),  IgA-TGA,  IgG & IgA antibodies to DGP,  IgA-anti-actin antibodies (IgA-AAA),  Villous-height to crypt-depth ratio (Vh:Cd),  IEL count | Mean serum iFABP significantly higher in Coeliac vs HC (mean [SD], Coeliac: 486±185 pg/mL vs HC: 290±135 pg/mL, P<0.001)  Serum iFABP levels were stable from 0d, 3d, and 7d of gluten challenge (mean [SD], 0d: 502±393 pg/mL, 3d: 613±337 pg/mL, 7d: 517±220 pg/mL)  Serum iFABP concentrations significantly increased following 14d gluten challenge (mean [SD], 0d: (502±393 pg/mL vs 14d: 918±778 pg/mL, P=0.001)  iFABP change during gluten challenge was similar in both the high- & low-dose gluten groups (P=0.304)  Serum iFABP concentrations increased from -14d to 14d 16 (80%) of patients with Coeliac  IgA-TGA and IgA/IgG-DGP titers increased from -14d to 14d in 11 (55%) & 14 (70%) patients with Coeliac, respectively  The degree of iFABP change during gluten challenge was not influenced by prior adherence length to GFD (P=0.898)  Serum iFABP did not significant decrease within 2wk of subsequent GFD following gluten challenge (mean [SD], 14d: 918±778 pg/mL vs 28d: 736±480 pg/mL)  At day 28, both TGA-IgA and DGP-IgA/IgG levels were elevated in 17 patients (85%, mean increase of 35.1±50.3 U/mL and 39.3±48.3 U/mL, respectively  Significant increase in IgA-AAA titers at 28d (mean [SD], 0d: 11.8±13.1 U/mL vs 28d: 15.2±15.1 U/mL, P=0.018)  IgA-AAA titers significantly increased from -14d to 14d in 11 (55%) patients and increased in 17 (85%) patients by 28d  Serum iFABP concentrations correlated significantly with IEL count both at -14d (r=0.458, P=0.042, n=20) & 14d of gluten challenge (r=0.654, P=0.002, n=19)  Serum iFABP change from -14d to day 14 correlated with IEL count both at -14d (rho=0.529, P=0.017, n=20) & 14d (rho=0.643, P=0.003, n=19)  Serum iFABP level at -14d showed a negative correlation trend with Vh:Cd at -14d (r=−0.425, P=0.062, n=20) and with Vh:Cd at day 14 (r=−0.447, P=0.055, n=19)  Serum iFABP increased significantly in 13/16 (81%) patients who experienced deterioration in Vh:Cd during gluten challenge |
| Bodelier 2016^[24]^ | Explore utility of iFABP in assessing disease activity in patients with existing Crohn’s & Colitis | Adults | Patients with endoscopy  Crohn’s: 59  Colitis: 11  Cross-sectional cohort  Crohn’s: 128  Colitis: 66 | Plasma iFABP (in-house ELISA),  SES-CD,  Mayo,  HBI,  SCCAI,  Faecal calprotectin  Active disease = FC>250 µg/g or a positive clinical activity index in combination with CRP>5 mg/l  Remission = negative clinical activity index, CRP< 5 mg/l, and FC<100 µg/g | Patients with endoscopy  25 (42%) patients with Crohn’s & 5 (45%) patients with Colitis had active disease based on endoscopic score  No significant difference in median plasma iFABP concentrations between Crohn’s patients with active disease vs remission (median [Q1, Q3], Crohn’s patients, active disease: 139.2 pg/mL [Q1: 103.3, Q3: 211.6] vs Crohn’s patients, remission: 119.2 pg/mL [Q1: 74.1, Q3: 197.8], P=0.37)  No significant difference in median plasma iFABP concentrations between patients with Colitis with active disease vs remission (median [Q1, Q3], Colitis patients, active disease: 107.8 pg/mL [Q1: 65.4, Q3: 159.2] vs Colitis patients, remission: 151.8 pg/mL [Q1: 111.1, Q3: 579.4], P=0.33)  iFABP did not significantly correlate with SES-CD (r=0.06; P=0.63) or with the Mayo score (r=−0.40; P=0.29)  Cross-sectional cohort  42% (54/128) of Crohn’s & 39% (26/66) of Colitis patients had active disease  iFABP levels did not significantly differ between disease locations in Crohn’s (P=0.79) or disease extension in Colitis (P=0.40)  No significant difference in median iFABP concentrations in patients with Crohn’s between active disease vs remission (median [Q1, Q3], Crohn’s patients, active disease: 145.5 pg/mL [Q1: 101.7, Q3: 227.0] vs Crohn’s patients, remission: 157.5 pg/mL [Q1: 105.5, Q3: 254.5, P=0.29)  Median iFABP was significantly higher in Colitis patients in remission vs active disease (median [Q1, Q3], Colitis patients, remission: 172.0 pg/mL [Q1: 108.1, Q3: 241.0] vs Colitis patients, active disease: 106.8 pg/mL [Q1: 73.4, Q3: 193.2], P=0.03)  iFABP did not differ between based on disease extension in patients with active Colitis (P=0.53)  iFABP did not differ between based on disease location in patients with active Crohn’s (ileal vs colonic disease P=0.83; ileal vs ileocolonic disease P=0.87)  No significant correlation between iFABP and FC, CRP, or clinical activity indices in patients with Crohn’s  Follow-up samples available from 18 patients with Crohn’s. Median follow-up time: 4 months (Q1: 2, Q3: 9). No significant difference in iFABP levels between patients in remission vs active disease (median [Q1, Q3], remission: 147.7 pg/mL [Q1: 107.8, Q3: 187.4] vs active disease: 121.4 pg/mL [Q1: 88.4, Q3: 250.7], P=0.78) |
| Sarikaya et al 2015 ^[25]^ | Explore iFABP concentrations between patients with Crohn’s and Controls | Adults | Active Crohn’s: 41  Remission Crohn’s: 33  Controls: 37 | Serum iFABP (Hycult Biotech),  CDAI,  Complete blood count,  ESR,  CRP,  Serum biochemistry | Serum iFABP significantly higher in patients with active Crohn’s vs remission Crohn’s (P=0.012) & Controls (P=0.038)  No statistical difference in serum iFABP concentrations between remission Crohn’s vs Controls (P=0.145)  Serum iFABP positively correlated with CDAI (r=0.319, P=0.006), & CRP (r=0.253, P=0.008)  Serum iFABP decreased significantly when patients with active Crohn’s entered clinical remission (P<0.001) |
| Gross et al 2015^[26]^ | Examine serum iFABP concentrations between patients with refractory Coeliac (RCoeliacII), Controls, ACD, and patients with Coeliac on GFD | Adults | RCoeliacII: 33,  Controls: 27,  Coeliac: 37  Coeliac GFD: 33 | Serum iFABP (in-house ELISA),  IEL count,  Marsh grading | Patients with RCoeliacII had significantly higher serum iFABP concentrations vs Controls, Coeliac GFD (median [Q1, Q3], RCoeliacII: 870 pg/mL [106, 2,234] vs Controls: 229 pg/mL [85, 1,338], P=0.001; vs Coeliac GFD: 170 [63, 1,572], P=0.003)  No significant difference in serum iFABP concentrations between patients with RCoeliacII vs Coeliac (median [Q1, Q3], RCDII vs Coeliac: 646 [113, 3,000])  Serum iFABP concentrations significantly higher in patients with villous atrophy (March IIIA-IIIC) (P<0.0001)  ROC curve analysis of using serum iFABP concentrations to aid discrimination between RCoeliacII vs Coeliac GFD had area-under-the-curve (AUC)=0.8 (P<0.0009) when using cut-off of 660 pg/mL with highest specificity (94%), sensitivity of 69%  Combined abnormal luminal pancreatic protein GP2A levels or abnormal serum iFABP concentrations improved ROC model, with sensitivity of 80% and specificity of 89% (AUC=0.82, P=0.02) |
| Bottasso Arias et al 2015^[27]^ | Investigate iFABP concentrations between patients with Coeliac, Controls and IBD | Adults & paediatrics | Controls: 42 (16 paediatric, 26 adults)  Newly diagnosed Coeliac: 40 (17 paediatric, 23 adults)  Coeliac following GFD: 9 (3 paediatric, 6 adults),  IBD: 7 (1 paediatric, 6 adults) | Serum iFABP (Hycult Biotech),  IgG & IgA anti-TGA,  IgG & IgA anti-DGP antibodies  iFABP, L-FABP mRNA expression (qPCR) | Increased mean serum iFABP concentration in patients with Coeliac patients at diagnosis (mean [range], Coeliac diagnosis: 2,898.89 pg/mL [616.83–7,295.95] vs non-Coeliac controls: 1,356.49 pg/mL [256.51–3,433.33], P<0.0001; vs Coeliac patients on GFD: 1,766.84 pg/mL [391.42–3,955.88], P=0.0002; vs IBD patients: 744.92 pg/mL [165.89–1,558.2 pg/mL], P=0.0264)  No significant difference in serum iFABP between patients with Coeliac following GFD vs non-Coeliac controls  No significant difference in serum iFABP between patients with IBD vs non-Coeliac controls  Adult non-Coeliac controls had significantly higher mRNA expression in the small intestine for both L-FABP and IFABP compared with paediatric  Expression of L-FABP & IFABP was significantly lower in untreated adult Coeliac patients compared with non-Coeliac control (LFABP P<0.0001, iFABP P=0.0423)  mRNA levels of both L-FABP and IFABP significantly higher in samples with severe enteropathy compared with healthy tissue |
| Adriaanse et al 2013 ^[17]^ | Explore relationship between serum iFABP concentrations and villous atrophy in patients with existing Coeliac | Adults | Coeliac: 80,  Controls: 141 | Serum iFABP (in-house ELISA),  Serum IgA-EMA,  Serum IgA-TGA,  Marsh grade | Serum iFABP signiﬁcantly elevated Coeliac vs Controls (median [Q1, Q3] Coeliac: 691 pg/mL [447, 1,266] vs Controls: 178 pg/mL [126, 286], p<0.001)  Serum iFABP concentrations increased concordant with villous atrophy severity (median [Q1, Q3] Marsh IIIA: 530 [384, 836], Marsh IIIB: 755 [475, 1,248], Marsh IIIC: 860 [556, 1,620] vs Controls, all P<0.001)  iFABP concentrations correlated signiﬁcantly with Marsh grade (r=0.265, P<0.05)  At Coeliac diagnosis, serum iFABP correlated significantly with IgA-TGA levels (r=0.403, P<0.01)  At 6mo GFD serum iFABP decreased significantly from diagnosis (P<0.01)  Serum iFABP remained significantly elevated during 1-2 yrs GFD vs HC (P<0.05)  Signiﬁcant correlation between serum iFABP Marsh grade during GFD (r=0.405, P<0.001)  Serum iFABP levels did not correlate with IgA-TGA during GFD (R=0.259, P=0.23) |
| Vreugdenhil et al 2011^[18]^ | Explore correlation between iFABP and villous atrophy in patients with Coeliac compared with HC | Paediatrics | Coeliac: 49,  Controls: 19 | Serum iFABP (Hycult Biotech),  IgA-TGA,  IgA-EMA,  Marsh Grade | Serum iFABP concentrations increased in patients with Coeliac compared with Controls (mean [range] Coeliac: 458 pg/ml [20–2970] vs HC: 20 pg/ml [20–485], P<0.001)  ROC analysis found iFABP concentration of 224 pg/ml optimal cut-off to discriminate Coeliac vs Controls (AUC: 0.92 [95% CI: 0.84–0.99], p<0.001)  iFABP concentrations normalised at 3 months GFD in 16/20 (80%) patients who had raised iFABP at diagnosis  iFABP decreased to below cut-off level in 23/25 (92%) patients within 26 weeks of GFD |
| Wiercinska-Drapalo et al 2008^[19]^ | Explore utility of iFABP with marker of intestinal injury in patients with existing Colitis | Adults | Colitis: 42,  Controls: 20 | Serum iFABP (Hycult Biotech),  CRP,  ESR,  White blood cell count,  Platelet count,  Haemoglobin,  Fibrinogen,  Total protein,  Albumin | Mean serum iFABP significantly increased in patients with Colitis vs HC (mean [SD], Colitis: 166.9± 36.3 vs. Controls: 61.3± 7.8 pg/mL, p<0.05)  Patients with pancolitis had increased iFABP vs left-sided colitis (mean [SD], pancolitis: 249.89±54.84 pg/mL vs left sided colitis: 61.8±8.5 pg/mL, p<0.001)  Patients with severe disease had increased serum iFABP concentration vs patients with mild disease (mean [SD], severe disease: 260.5±60.6 vs mild disease: 61.5±7.9 pg/mL, p<0.001)  Serum iFABP strongly significantly correlated with Meyers scale (r=0.86, p<0.001)  Serum iFABP moderately significantly correlated with hsCRP (r=0.33, p<0.05)  No significant correlation between serum iFABP and patients age, sex, disease duration, or with routine laboratory parameter |
| Abbreviations: Coeliac: coeliac disease, Controls: healthy controls, Crohn’s: Crohn’s disease, UC: Colitis, CDAI: Crohn’s Disease Activity Index, IGF: insulin-like growth factor, CLDN-3: claudin 3, TGA: tissue transglutaminase, HLA: human leukocyte antigen, DGP: deamidated gluten peptides, ULN: upper limit of normal, EMA: endomysial antibodies, ELISA: enzyme-linked immunosorbent assay, qPCR: quantitative polymerase chain reaction. iFABP: intestinal fatty acid binding protein, IFX infliximab, IL: interleukin, TNF: tumour necrosis factor, CRP: C reactive protein, ESR: erythrocyte sedimentation rate, HBI: Harvey-Bradshaw index, SD: standard deviation, ROC: receiver operating characteristic, wk: weeks, NCWS: non-Coeliac wheat sensitivity, LPS: lipopolysaccharide, LBP: LPS-binding protein, SES-CD: simple endoscopic score, SCCAI: simple clinical colitis activity index, FC: faecal calprotectin, L-FABP: liver fatty acid binding protein, villous-height to crypt-depth ratio (Vh:Cd), intraepithelial lymphocyte count (IEL), AUC: area-under-the-curve, RCoeliacII: refractory celiac disease type II | | | | | |
